# Housing and Stroke Outcomes: Why Aging in Place Needs to Be Equitable

**DOI:** 10.3390/ijerph23070824

**Published:** 2026-06-23

**Authors:** Roshanak Mehdipanah, Leanna Delhey, Fatema Shafie Khorassani, Sehee Kim, Xu Shi, Lewis B. Morgenstern, Erin Case, Lynda D. Lisabeth

**Affiliations:** 1 Department of Health Behavior and Health Education, School of Public Health, University of Michigan, Ann Arbor, MI 48109, USA; 2 Department of Epidemiology, School of Public Health, University of Michigan, Ann Arbor, MI 48109, USA; 3 Department of Biostatistics, School of Public Health, University of Michigan, Ann Arbor, MI 48109, USA; 4 Department of Clinical Epidemiology and Biostatistics, University of Ulsan College of Medicine, Asan Medical Center, Seoul 05505, Republic of Korea; 5 Department of Neurology, University of Michigan Medical School, Ann Arbor, MI 48109, USA

**Keywords:** stroke recovery, housing conditions, Hispanic communities, neighborhood effects

## Abstract

**Highlights:**

**Public health relevance—How does this work relate to a public health issue?**

- It examines disparities in post-stroke outcomes in ethnically diverse neighborhoods, focusing on Hispanic populations in a bi-ethnic U.S. community.
- It investigates neighborhood housing values as a proxy for housing conditions and their relationship to stroke recovery outcomes.

**Public health significance—Why is this work of significance to public health?**

- It identifies that higher neighborhood Hispanic composition is associated with poorer post-stroke outcomes, highlighting persistent health inequities.
- It demonstrates that housing value alone does not explain these disparities, suggesting more complex structural and social determinants of health.

**Public health implications—What are the key implications or messages for practitioners, policy makers and/or researchers in public health?**

- We emphasize the need to incorporate individual-level housing conditions and environmental factors in stroke recovery research and interventions.
- We support the development of targeted, equity-focused public health strategies to improve post-stroke outcomes in minoritized communities.

**Abstract:**

Limited research on the impacts of housing conditions on post-stroke outcomes exists, particularly in minoritized neighborhoods that face greater housing disparities than non-Hispanic White neighborhoods. This is largely due to the unavailability of data that combines residential conditions and post-stroke outcomes in diverse communities. This brief report explored whether neighborhood housing values, a proxy of housing conditions, mediate the association between neighborhood ethnic composition (%Hispanic) and post-stroke outcomes in Nueces County, a bi-ethnic community in south Texas. Linear regression models were used to assess associations adjusted for socio-demographics, pre-stroke factors and stroke severity. A total of 782 ischemic stroke cases in 78 neighborhoods were included. Greater %Hispanic was associated with worse post-stroke outcomes and lower housing values. Higher housing values did not change the association between %Hispanic and post-stroke outcomes. Future studies should consider including individual-level data on housing conditions and the capacity to make modifications in research related to post-stroke recovery.

## 1. Introduction

Stroke is the leading cause of adult disability in the U.S., but risks associated with stroke incidence and recovery vary with race and ethnicity [1]. Mexican Americans (MAs) are the largest Hispanic sub-group in the U.S., comprising 63% of the Hispanic American (50.5 million) population [2]. Prior research has consistently shown disparities in stroke incidence, recovery, and long-term disability among MA populations compared with non-Hispanic Whites (NHWs) [3,4]. A 2014 study by Lisabeth and colleagues showed that MAs scored worse than non-Hispanic whites (NHWs) on neurological, functional and cognitive outcomes after adjustment for confounding factors [5]. Research among MA stroke survivors has also reported poorer access to physician care and medications compared to NHW stroke survivors, contributing to disparities in stroke recovery and adaptive capacity following stroke [6].

Beyond healthcare access, living conditions and neighborhood environments may substantially shape recovery after stroke. Recovery often requires housing environments that can accommodate mobility limitations and home modifications needed for independent living. However, few studies have connected the associations between housing conditions and post-stroke recovery [7,8], and none to our knowledge have examined ethnic differences despite well-documented racial and ethnic housing inequities [9].

Hispanic populations, including MAs, are more likely to live in socioeconomically disadvantaged neighborhoods characterized by poverty, limited resources, and poorer housing quality [10,11]. These conditions may influence post-stroke recovery through differences in environmental safety, accessibility, social support, and the ability to modify homes for disability-related needs [12,13]. Although homeownership among Hispanic populations is increasing, rates of homeownership and housing values of those homes are far lower than for NHWs [14]. Higher housing values are associated with better quality homes, including those that are more likely to accommodate modifications if needed. In a study by Ryu and colleagues (2017), factors like older age of the house and poorer quality and location, all factors influencing housing values, were associated with increased risk of falls requiring emergency care [15]. Research also shows that homeowners, compared to renters, are more likely to make repairs and modifications to their homes as needed, not only due to higher disposable incomes, but also due to additional barriers renters may experience [9]. Such barriers include the need to find homes that are already accessible or landlords that are willing to make those repairs and modifications. In some situations, renters are often economically responsible for those changes as well as the potential costs of restoring the home to its original state upon moving, adding more burden. These structural and environmental factors may contribute to disparities in recovery and long-term functional outcomes after stroke.

To address gaps in the existing research, we used neighborhood- and individual-level data to examine whether neighborhood Hispanic composition is associated with post-stroke functional outcomes and whether neighborhood housing values mediate this association. Nueces County, Texas, where 63.7% of residents identify as Hispanic and 59.3% as MA, of whom 92% are US born, provides an important setting for examining neighborhood- and housing-related contributors to ethnic disparities in stroke recovery [16].

## 2. Methods

This study used data from the Brain Attack Surveillance in Corpus Christi (BASIC) Project for years 2012–2016 and the American Community Survey (ACS) 2012–2016. These datasets were selected because BASIC provides population-based stroke surveillance data with detailed individual-level clinical and post-stroke functional outcome information, while the ACS provides standardized neighborhood-level socioeconomic and housing measures that can be linked at the census tract level. Briefly, the BASIC project was established in January of 2000 and is an ongoing study using active and passive surveillance methods where all cerebrovascular events, including ischemic stroke and intracerebral hemorrhage, among Nueces County, Texas, residents are recorded [17]. The 2012–2016 period represented the most recent years for which linked individual-level stroke outcome data and harmonized neighborhood-level ACS measures were available for this analysis at the time of study development. Additionally, the use of 5-year ACS estimates provided a more stable and precise value for our census-tract-level socioeconomic and housing measures, particularly in smaller geographic areas. A more detailed description of our methods, including sampling process and data collection, can be found in ref. [17]. The BASIC study was approved by the institutional review board of the University of Michigan and those of the participating hospitals.

### 2.1. Study Sample

Participants were recruited from the BASIC project aged 45 years or older, with complete 90-day post-stroke health outcome data. Eligible participants were Nueces County residents with confirmed cerebrovascular events identified through the BASIC project’s active and passive surveillance system. Using geocoded home address data from medical records, participants were assigned to a census tract in the county. Census tracts were used as a proxy for neighborhoods and were the finest spatial scale at which all data used in the analysis were available. Those who did not have a complete address were excluded.

### 2.2. Dependent Variables

Activities of Daily Living (ADL) and Instrumental Activities of Daily Living (IADL) scales were used to measure post-stroke functional outcomes. The ADL scale measures seven basic functional abilities (walking, bathing, grooming, eating, dressing, moving, toileting), and the IADL scale includes fifteen questions related to daily functioning. For both ADL and IADL outcomes, participants self-reported the level of difficulty performing each ADL/IADL activity by themselves on a scale of 1 to 4: 1 (no difficulty), 2 (some difficulty), 3 (a lot of difficulty), and 4 (can only do with help). For ease of interpretation, responses were summed for each of the 22 ADL/IADL items and divided by the number of items for an average score ranging from 1 to 4, to match the original scales [5].

### 2.3. Independent Variables

Neighborhood-level percent Hispanic composition and median housing values were derived from the ACS 2012–2016 5-year estimates [18]. Neighborhood housing value was used as a proxy measure for neighborhood housing conditions because direct measures of individual housing quality were unavailable. Prior research has shown that housing values are associated with multiple dimensions of neighborhood residential environments, including housing quality, maintenance, and investment, while also reflecting neighborhood amenities and built environment characteristics [19]. Housing conditions play a substantial role in the appraisal process [20,21]. However, while we recognize that housing values do not directly measure physical housing conditions and may be influenced by factors such as location and housing market dynamics, they have been used in neighborhood and housing research as neighborhood-level indicators of housing and neighborhood context when direct housing quality measures are unavailable [22,23,24].

### 2.4. Covariates

Models were adjusted for neighborhood-level age (%<18, %18–65, %65+), gender (%female), marital status (%married), education (%no education, %high school or more), and %non-homeowners, all obtained from the ACS. Except for model 2, all models were also adjusted for individual-level socio-demographic characteristics, including age (57–63, 64–72, 73+; based on sample distribution), sex, race–ethnicity (MA, NHW), education (less than high school, high school, college or more), marital status (single/never married, married/living with someone, widowed, divorced/separated), and health insurance (yes/no). Models were also adjusted for pre-stroke and stroke-related factors, including National Institutes of Health Stroke Score (NIHSS; mild, moderate and severe), Informant Questionnaire for Cognitive Decline in the Elderly (IQCODE; normal, cognitive impairment no dementia, dementia), modified Rankin scale (no disability, slight to moderate disability, severe disability), and comorbidity score.

### 2.5. Data Analysis

We used a mediation analysis framework to sequentially assess whether housing value mediates the association between ADL/IADL scores and the percent of Hispanic residents (%Hispanic) in a neighborhood. Conceptually, we hypothesized that neighborhoods with more Hispanic residents may have lower neighborhood housing values, reflecting differences in neighborhood housing and socioeconomic conditions, which may subsequently influence post-stroke functional outcomes. To test this, the analysis considered three models. In model 1, we examined the individual-level association between each post-stroke outcome and neighborhood %Hispanic. In model 2, we examined the neighborhood-level association between neighborhood housing values and %Hispanic. In model 3, we built on model 1 by adjusting for neighborhood housing values. The indirect effect of housing value was further assessed using the Sobel test. This mediation framework follows a traditional regression-based approach, supplemented with Sobel testing [25]. We acknowledge that mediation analyses rely on assumptions, including no unmeasured confounding of the exposure–outcome, exposure–mediator, and mediator–outcome relationships, as well as correct model specification [26].

All models were fit using linear regression, with each post-stroke outcome as the dependent variable, and generalized estimating equations were used in the individual-level analyses to account for the clustering of subjects within neighborhoods. Missing individual-level data were handled by multiple imputations and inverse probability weighting. A sensitivity analysis was done to compare imputed data with the complete data analysis results, and no significant differences were noted. We assessed the functional form of the continuous variables included in the model and log-transformed housing value, which was heavily right-skewed. We re-scaled %Hispanic and %non-homeowners by their inter-quartile ranges (IQRs), so that a one-unit change in either variable can be interpreted as a comparison between the 75% and 25% quartiles.

## 3. Result

A total of 782 BASIC participants were included in the study, living in 78 neighborhoods in Nueces County. Descriptions of the individual- and neighborhood-level characteristics are provided in Table 1. Briefly, most participants were MA (62.3%), had a high school or higher education (71.7%), had health insurance (84.6%), and had a mild stroke (77.5%) according to the NIHSS. The median ADL/IADL score was 1.82 (IQR: 1.26–2.50), representing mild disability. At the neighborhood level, the median of percent Hispanic residents was 62.7% (IQR: 47.9–80.3), and the median housing value was 108,550 (IQR: 67,900–147,000).

In model 1 (Table 2), an IQR increase in %Hispanic was significantly associated with a worse ADL/IADL score (0.175 mean change, *p* < 0.001). In model 2, an IQR decrease in %Hispanic was also significantly associated with lower housing values (−0.152 mean change, *p* < 0.01). In model 3, IQR increases in %Hispanic residents remained significantly associated with ADL/IADL score (0.172 mean change, *p* < 0.01), while housing values were not significantly associated (−0.007 mean change, *p* > 0.05). The addition of housing value to the model did not alter the association between percent Hispanic residents and ADL/IADL score, indicating no mediation effect.

## 4. Discussion

Our study showed that neighborhoods with a higher percentage of Hispanic residents were associated with worse post-stroke functioning measured by ADLs/IADLs, even after adjusting for a comprehensive set of individual-level confounders. No significant association existed between housing values and ADL/IADL scores when added to the adjusted model for the association between the percentage of Hispanic residents and post-stroke functional outcomes. Therefore, neighborhood housing values do not appear to mediate this relationship. However, the percentage of Hispanic residents in the neighborhood and housing values were negatively associated. This provides some insight into existing housing inequities among Hispanics in Nueces County and their potential implications on post-stroke outcomes as described below.

This research contributes to existing work, including that completed in the BASIC project, establishing the relationship between neighborhood factors like socioeconomic status, income, cohesion, and ethnic composition, and their associations with stroke prevalence, recovery, and outcomes [27,28,29]. Yet, this is the first study of its kind, to our knowledge, to examine a neighborhood-level housing factor and its association with stroke outcomes in the U.S., and one of the few examining housing conditions as a determinant of post-stroke recovery. Prior research looking at perceived measures of housing conditions and their impact on stroke recovery have relied on sample sizes of less than 20 [30], while another study modeled the association of excess stroke death risk on age of housing and access to heating, showing positive associations [31]. Although housing values were not found to mediate the association between percentage Hispanic residents and post-stroke recovery, our study did find that neighborhoods with higher proportions of Hispanic residents were associated with lower housing values. These findings are in line with a recent study showing that homes in NHW neighborhoods appreciated $200,000 more than comparable homes in similar communities of color from 1980 to 2015 in the U.S. [32].

### Limitations

Several limitations should be considered when interpreting these findings. First, this study is observational in nature, and the results should be interpreted as correlational rather than causal. Although we adjusted for a comprehensive set of individual-level confounders, residual confounding from unmeasured factors may remain. Second, the neighborhood-level associations observed should not be interpreted as causal effects at the individual level. Our analyses used aggregated neighborhood measures, and findings reflect contextual patterns and may be subject to ecological fallacy. Therefore, the observed cannot be assumed to apply to individual residents. Finally, we recognize the limitations of using housing values as a proxy for housing conditions, as housing values may reflect a combination of housing quality, neighborhood desirability, local amenities, and broader socioeconomic conditions rather than housing characteristics relevant to stroke recovery. Future studies should incorporate individual-level measures of housing quality and repair needs, dwelling age and type, accessibility features, unmet home modification needs, housing affordability, and residents’ ability and capacity to make modifications following stroke. Ideally, this data would be collected through additional survey questions and observational data collection by trained field staff.

## 5. Conclusions

This study contributes to the limited literature considering housing condition impacts on stroke recovery, using housing values as a proxy. Housing values did not mediate the relationship between percent Hispanic residents and post-stroke outcomes. However, our research did show potential ethnic disparities in housing, and further research with housing-specific data at the individual level is needed to understand how housing conditions can impede successful post-stroke recovery at home and contribute to ethnic stroke outcome disparities.

## Figures and Tables

**Table 1:** Description of individual- and neighborhood-level characteristics.

	Sample (N)	Median (IQR) OR N (%)
***Individual-level characteristics***		
**Age**	782	
<57		185 (23.7%)
57–63		182 (23.3%)
64–72		208 (26.6%)
73+		207 (26.5%)
**Gender**	782	
Female		367 (46.9%)
**Race–ethnicity**	782	
Mexican American		487 (62.3%)
Non-Hispanic White		295 (37.7%)
**Education**	781	
Less than high school		221 (28.3%)
High school		227 (29.1%)
College or more		333 (42.6%)
**Marital status**	781	
Single/never married		73 (9.4%)
Married/living with someone		387 (49.6%)
Widowed		156 (20.0%)
Divorced/separated		165 (21.1%)
**NIHSS**	779	
Mild		604 (77.5%)
Moderate		175 (22.5%)
**IQCODE**	651	
Normal		381 (58.5%)
Cognitive impairment no dementia		200 (30.7%)
Dementia		70 (10.8%)
**MRS**	766	
No disability		418 (54.6%)
Slight to moderate disability		310 (40.5%)
Severe disability		38 (5.0%)
**Comorbidity score**	782	2 (1, 4)
**Health insurance**	765	
Yes		647 (84.6%)
No		118 (15.4%)
**ADL/IADL**	769	1.82 (1.23, 2.50)
***Neighborhood-level characteristics***		
Hispanic/Latinx residents (%)	78	62.70 (47.94, 80.28)
Median housing value ($)	78	108,550 (67,900, 147,000)
Homeownership (%)	78	61.17 (45.18, 72.55)

IQR: Inter-quartile range. Bolded/italicized words represent headings.

**Table 2:** Regression model results.

	ADL/IADL				Log (Housing Value)			
	Est	SE	95% CI	*p*-Value	Est	SE	95% CI	*p*-Value
Model 1								
IQR change in neighborhood %MA	0.18	0.04	0.09, 0.26	<0.001	-	-	-	
Model 2								
IQR change in neighborhood %MA	-	-	-		−0.54	0.05	−0.64, −0.44	<0.001
Model 3								
IQR change in neighborhood %MA	0.17	0.05	0.07, 0.27	0.001	-	-	-	
Log (housing values)	−0.01	0.09	−0.18, 0.17	0.934	-	-	-	
Sobel T Statistic	0.08	-	-	0.933				

IQR: Inter-quartile range; Est: estimate coefficient; SE: standard error; CI: confidence interval. Note: All models were adjusted for neighborhood-level IQR change in %renters and individual-level age, sex, race–ethnicity, education, marital status, health insurance, National Institutes of Health Stroke Score, Informant Questionnaire for Cognitive Decline in the Elderly score, modified Rankin scale score, and comorbidity score. Models 1 and 3: N = 782; model 2: N = 77.

## Data Availability

Access to the de-identified data that supports the findings of this study will be considered upon reasonable request to the corresponding author based on existing IRB approvals and data use agreements.
